# Endogenous auxin biosynthesis and *de novo* root organogenesis

**DOI:** 10.1093/jxb/erw250

**Published:** 2016-07-07

**Authors:** Ya Lin Sang, Zhi Juan Cheng, Xian Sheng Zhang

**Affiliations:** State Key Laboratory of Crop Biology, College of Life Sciences, College of Forestry, Shandong Agricultural University, Taian, Shandong 271018, China

**Keywords:** Adventitious root, auxin biogenesis, *de novo* organogenesis, plant regeneration, *YUCCA* family

**Induction of adventitious roots is essential for vegetative propagation of plants, and auxin has long been used as an exogenous root-inducing agent. In this issue of *Journal of Experimental Botany*, Chen *et al*. (pages 4273–4284) demonstrate that different members of the *YUCCA* family orchestrate the endogenous auxin biosynthesis that is required for the induction of adventitious roots.**

Sun Wukong, also known as the Monkey King, is the main character in the classical Chinese novel *Journey to the West*. As a fabled deity, he was endowed with magical properties allowing each of his hairs to be transformed into clones of himself as needed. Plants also possess the remarkable ability of multiplication, with detached pieces of adult tissues capable of forming an entire plant body (Gordon *et al.*, 2007; Birnbaum and Sánchez Alvarado, 2008; Sugimoto *et al.*, 2010). This unique ability is mainly based on *de novo* organogenesis, in which adventitious shoots or roots are generated from isolated tissues or organs (Duclercq *et al.*, 2011; Cheng *et al.*, 2013; Xu and Huang, 2014).

*De novo* organogenesis can be induced under both natural and tissue culture conditions (Chen *et al.*, 2014). Plant organs, such as stems and leaves, give rise to adventitious roots under natural growth conditions and this property has long been used for vegetative propagation of elite genotypes in agriculture, forestry and horticulture (De Klerk *et al.*, 1999). Six decades ago, Skoog and Miller demonstrated that the entire plant could be regenerated by tissue culture (Skoog and Miller, 1957). They showed that culturing explants in medium containing high levels of cytokinin induced the formation of adventitious shoots, whereas medium with high levels of auxin triggered initiation of adventitious roots. This classic system laid the foundations for plant micropropagation and genetic transformation (Duclercq *et al.*, 2011; Li *et al.*, 2011). In both cases, a key step ensuring the success of plant regeneration is *de novo* root organogenesis, which guarantees the water and nutrient supply for regeneration and survival of the new organism (Chen *et al.*, 2014).

**Box 1. Modulation of the dynamic expression pattern of *YUC4* in response to wounding**

After detachment, leaf explants were cultured on B5 medium without exogenous hormone. At day 0, *YUC4* is expressed in the hydathode. During the formation of adventitious roots, its expression is enhanced in mesophyll cells. After two days, strong expression signals are detected in the vascular tissues near the wound, where the adventitious roots initiate. DAC, days after culture.

**Figure:**
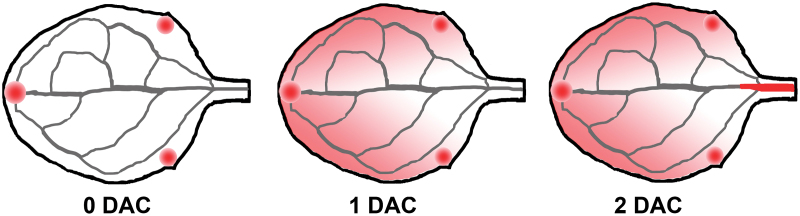

## Cell fate transition

The first cellular event of *de novo* organogenesis is cell fate transition (Duclercq *et al.*, 2011; Chatfield *et al.*, 2013). Evidence from different species has indicated that adventitious roots initiate from the procambium or cambium regions (Liu *et al.*, 2014; Xu and Huang, 2014). To study the underlying mechanisms, Chen *et al*. have developed a simple system to mimic the formation of adventitious roots under natural conditions (Chen *et al.*, 2014). By culturing Arabidopsis leaves on B5 medium without exogenous hormones for six to eight days, adventitious roots can be generated from the mid-vein near the wound (Liu *et al.*, 2014). Using this system, Liu *et al*. revealed that the cell fate transition during the initiation of rooting contains two steps. In the first step, *WUSCHEL-RELATED HOMEOBOX 11* (*WOX11*) and *WOX12* act redundantly to regulate the transition from procambium cells to root founder cells. In the second step, root founder cells are further switched into root primordium cells, marked by *WOX5* expression.

Notably, endogenous auxin plays essential roles in the cell fate reprogramming process. Inhibiting polar auxin transport using naphthylphthalamic acid (NPA) abolishes rooting, an effect which can be rescued by exogenous indole-3-acetic acid. During adventitious root formation, the distribution of auxin response signals overlaps with the expression regions of *WOX11*. Mutations of the auxin response elements within the *WOX11* promoter or NPA treatment disrupt the expression pattern of *WOX11* (Liu *et al.*, 2014). Moreover, the auxin response signals are progressively enhanced and distributed in the region of root initiation, suggesting that the wounding induces auxin accumulation in this area. However, the molecular events between explant detachment and adventitious root initiation remain to be elucidated.

## YUCCA enzymes

The research reported in this issue by Chen *et al.* (2016) describes the involvement of different members of the *YUCCA* (*YUC*) family, which encode enzymes catalysing the rate-limiting step of auxin biosynthesis in *de novo* root organogenesis. Under natural growth conditions, some plant species can generate adventitious roots from detached organs spontaneously, but in most cases application of exogenous auxin is required (De Klerk *et al.*, 1999), suggesting that the endogenous auxin biosynthesis varies among the different species. However, using culture methods to induce rooting using exogenous auxin could bypass the function of endogenous hormones. In the system used by Chen *et al.* (2016), no exogenous hormones were added. Thus, adventitious root generation depended on endogenous hormones, providing an opportunity to investigate the roles of endogenous auxin in *de novo* root organogenesis.

Together with the previous findings from this lab (Chen *et al.*, 2014; Liu *et al.*, 2014), the results support a working model for *de novo* root organogenesis (see Chen *et al.*, 2016, Fig. 9). The detachment of leaf explants leads to significant increases in the level of auxin. The elevated auxin content results from the function of *YUC* genes, which respond to wounding and act upstream of cell fate transition from competent cells (procambium and vascular parenchyma cells) to root founder cells. The *YUC* genes show division of labour and orchestrate auxin biosynthesis required for the formation of adventitious roots. Of them, *YUC1*, *2*, *4* and *6* play major roles under both light and dark conditions. *YUC1* and *4* function in a wounding-induced way, whereas *YUC2* and *6* contribute to the basal auxin level (Box 1). In addition, *YUC5*, *8* and *9* mainly produce auxin in leaf margin and mesophyll cells in response to darkness.

## More questions

A critical open question is how wounding signals trigger the spatial expression of *YUC1* and *4*. The authors suggest that wounding-response factors could regulate *YUC1* and *4* in cooperation with epigenetic factors. This is supported by the fact that up-regulation of *YUC1* and *4* expression is accompanied by a reduced level of histone H3 lysine 27 trimethylation, a marker of transcriptional repression (Schatlowski *et al.*, 2008; He *et al.*, 2013). It would be interesting to investigate the relationship between wounding signals and epigenetic factors, as well as their regulatory role in *de novo* root organogenesis.

Despite its importance to plant industries worldwide, adventitious root induction is still difficult in many species, hampering the development of forestry and horticulture (Rasmussen *et al.*, 2012). The issue is mainly limited knowledge about the mechanisms controlling adventitious root formation, and therefore the findings presented here provide valuable new information. Genetic engineering approaches allowing the modification of endogenous auxin biosynthesis would now be powerful in enhancing our abilities.
